# Supplementary material for: Microbiota Influences Fitness and Timing of Reproduction in the Fruit Fly Drosophila melanogaster
Source: Microbiol Spectr. 2021 Sep 29;9(2):e00034-21. doi: 10.1128/Spectrum.00034-21 (PMC8557915; doi:10.1128/Spectrum.00034-21)
Supplement: SUPPLEMENTAL FILE 1 — Supplemental material. Download SPECTRUM00034-21_Supp_1_seq7.pdf, PDF file, 1.8 MB [file spectrum00034-21_supp_1_seq7.pdf]

Supplemental figures

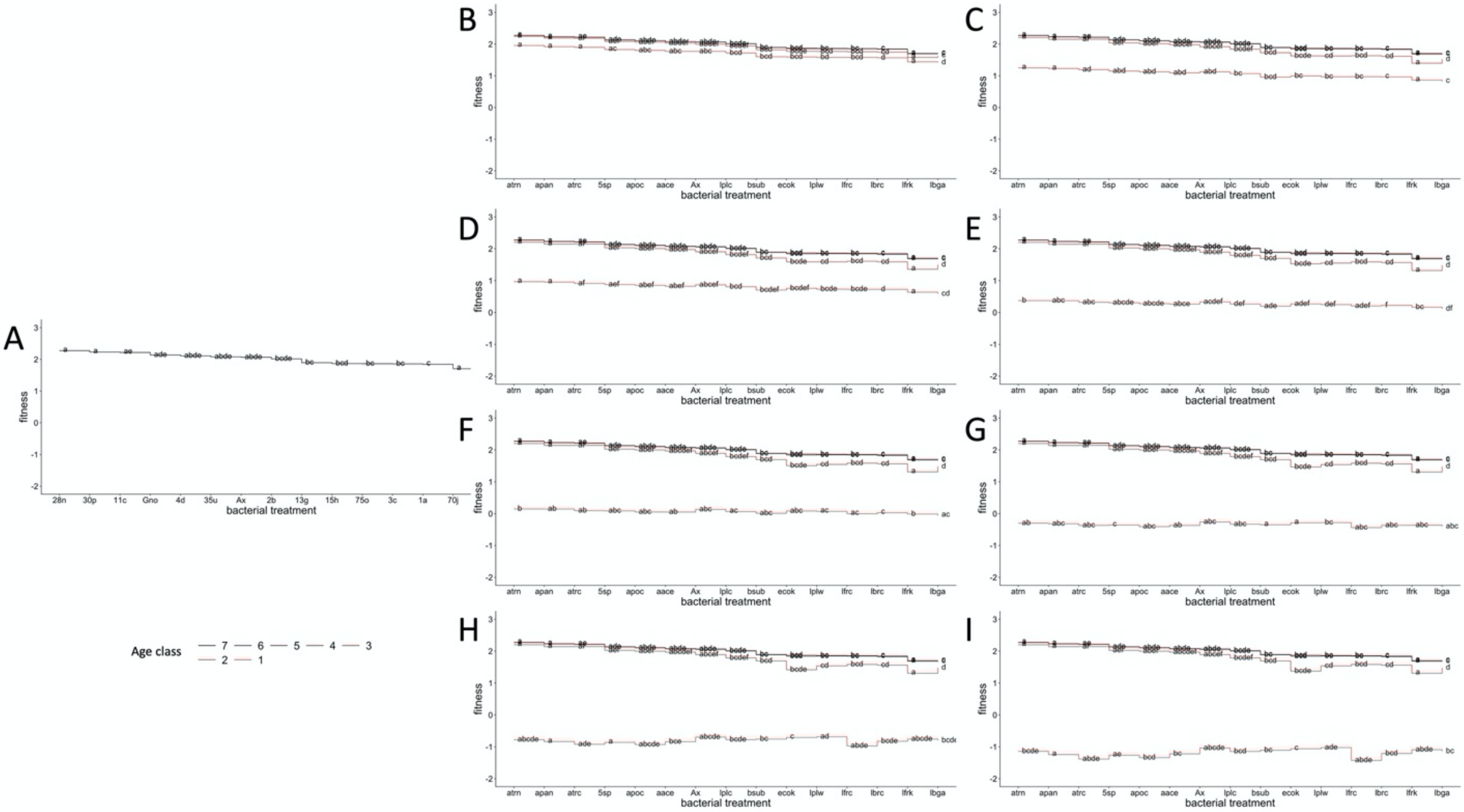

Figure S1. The effect on fly fitness of permuting fly survival rates under different bacterial treatments. To permute fly fitness, the same denominator was applied to the observed survival of a single age class across all of the different bacterial treatments. In each panel, permutations for all 7 age classes, all using the same denominator, are shown. Each different panel represents a different denominator: A) 1 (no permutation); B-I) 2, 10, 20, 100, 200, 1000, 10000, 100000. Each age class is shown as a distinct shaded line (see legend to match age class number with shading). The

values are not continuous between treatments, but a single line facilitates visualizing the changes in rank order of fitness values conferred by the different bacterial treatments when permuted. The average eigenvalue lambda is shown on the y-axis and is log-transformed. Different bacterial treatments are shown on the x-axis, using 4 letter codes from table 2. Data from vials containing flies reared with *P. putida*, which had characteristics of a fly pathogen, were omitted from these analyses.

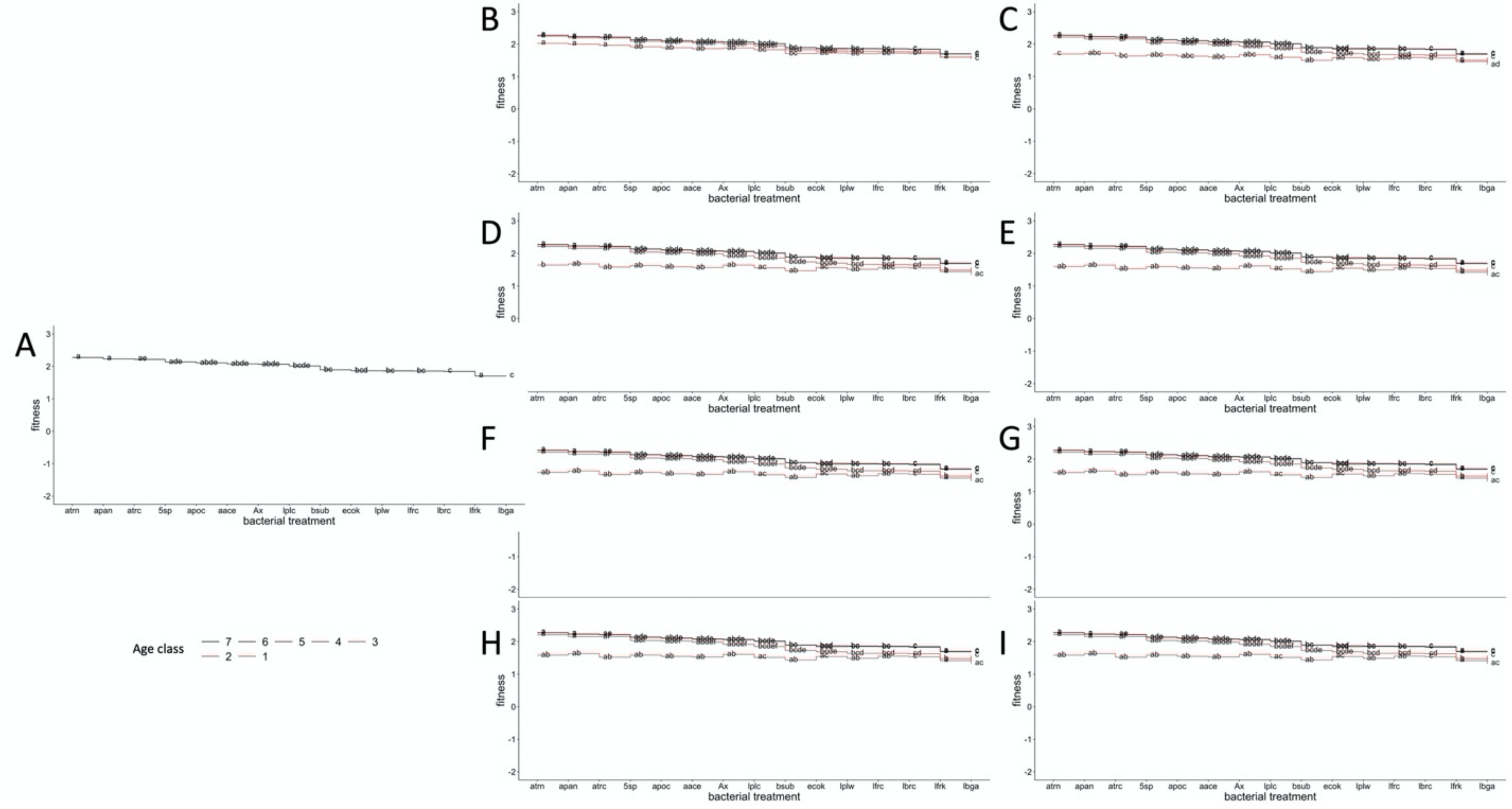

Figure S2. The effect on fly fitness of permuting fly fecundity under different bacterial treatments. To permute fly fecundity, the same denominator was applied to the observed fecundity of a single age class across all of the different bacterial treatments. In each panel, permutations for all 7 age classes, all using the same denominator, are shown. Each different panel represents a different denominator: A) 1 (no permutation); B-I) 2, 10, 20, 100, 200, 1000, 10000, 100000. Each age class is shown as a distinct shaded line (see legend to match age class number with shading). The values are not continuous between treatments, but a single line facilitates visualizing the changes in rank order of fitness values conferred by the

different bacterial treatments when permuted. The average eigenvalue lambda is shown on the y-axis and is log-transformed. Different bacterial treatments are shown on the x-axis, using 4 letter codes from table 2. Data from vials containing flies reared with *P. putida*, which had characteristics of a fly pathogen, were omitted from these analyses.

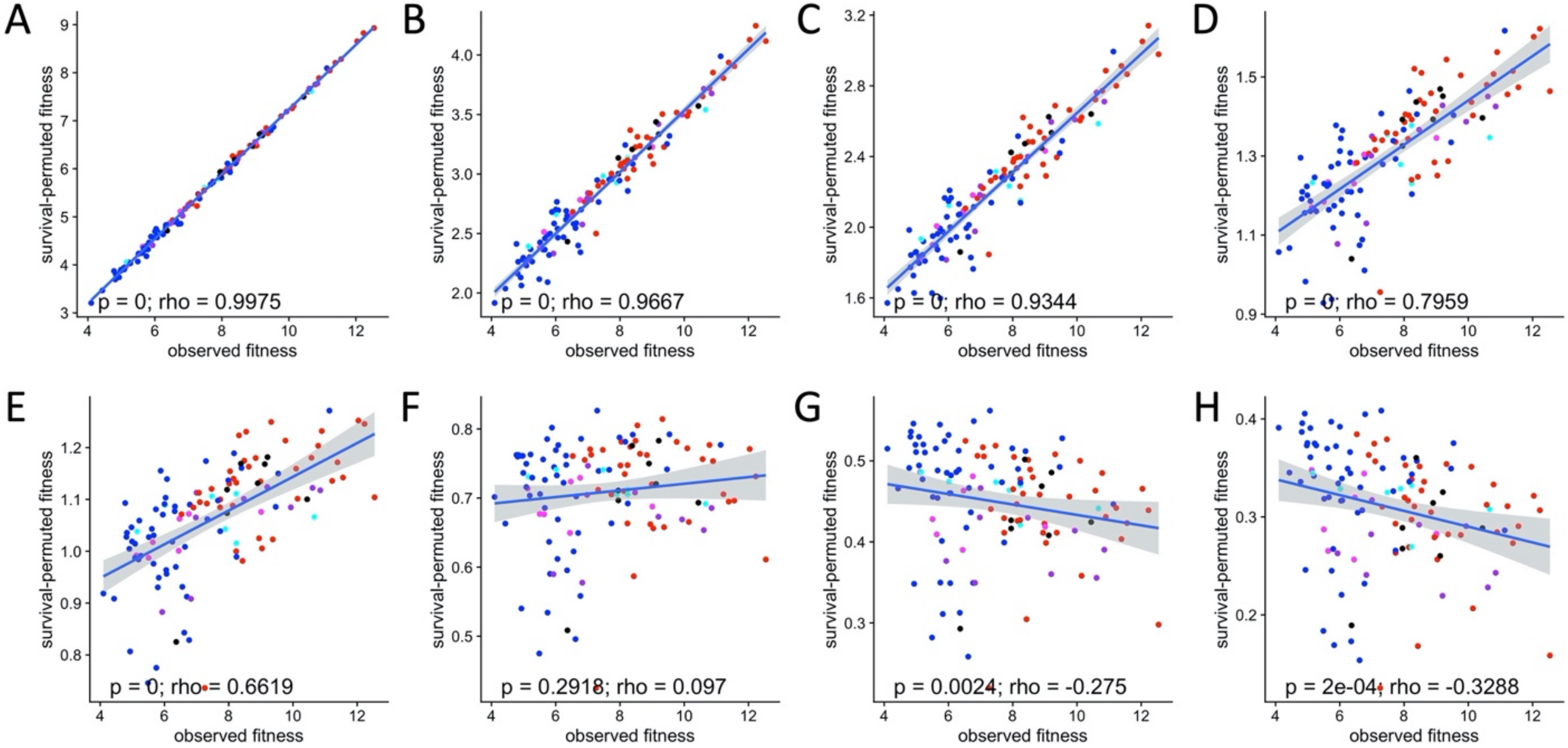

Figure S3. The effect on fly fitness of permuting fly survival rates under different bacterial treatments. To permute fly fitness, the same denominator was applied to the observed survival of a single age class across all of the different bacterial treatments. Each panel compares for each vial the observed fitness values against fitness when survival was permuted in the first age class by a different denominator: A-H) 2, 10, 20, 100, 200, 1000, 10000, 100000. Dot color indicates high-taxonomic level classifications for the treatments: red (AAB), blue (LAB), magenta (*B. subtilis*), cyan (Enterobacteriales), purple (5-sp), black (axenic). Data from vials containing flies reared with *P. putida*, which had characteristics of a fly pathogen, were omitted from these analyses.

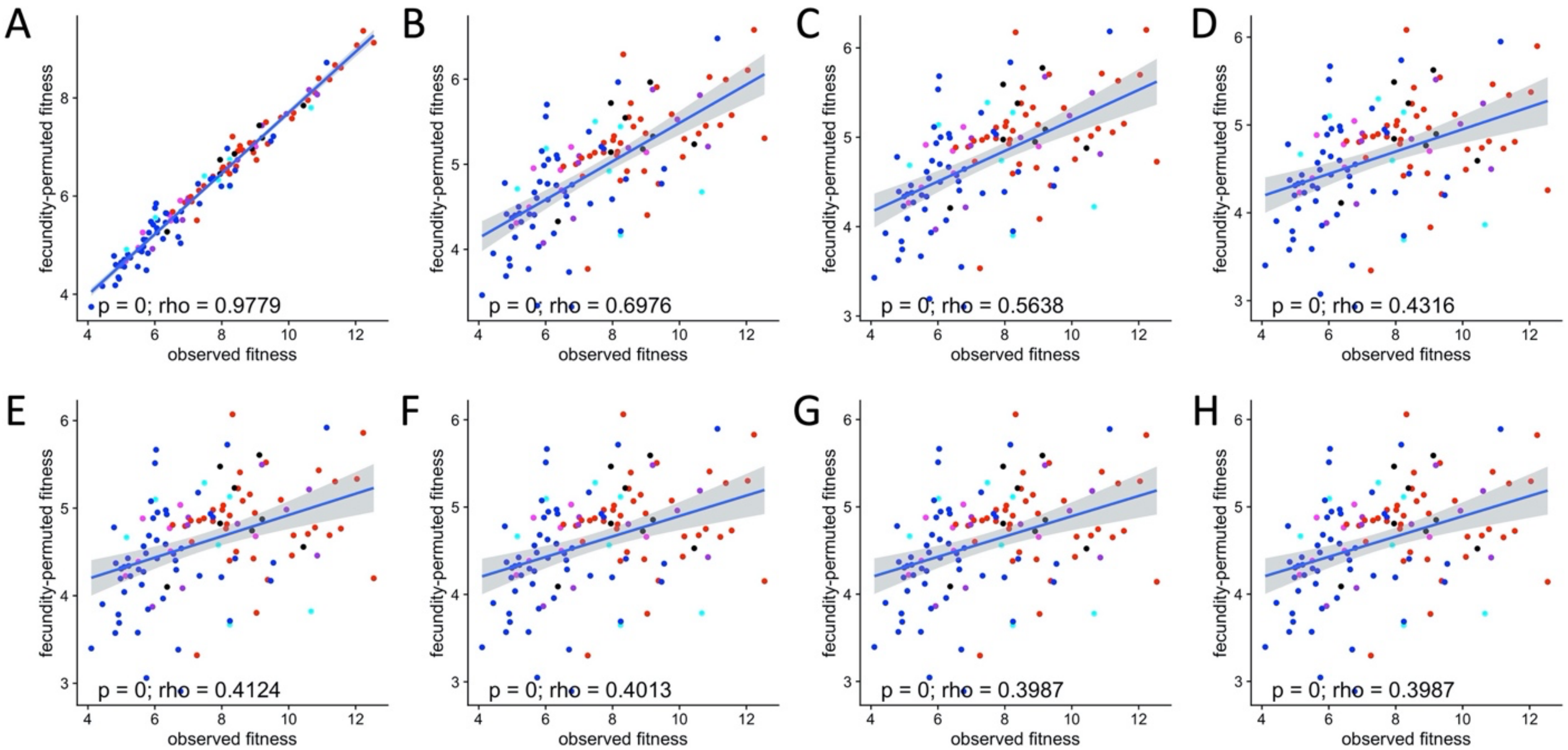

Figure S4. The effect on fly fitness of permuting fly fecundity under different bacterial treatments. To permute fly fitness, the same denominator was applied to the observed fecundity of a single age class across all of the different bacterial treatments. Each panel compares for each vial the observed fitness values against fitness when fitness was permuted in the first age class by a different denominator: A-H) 2, 10, 20, 100, 200, 1000, 10000, 100000. Dot color indicates high-taxonomic level classifications for the treatments: red (AAB), blue (LAB), magenta (*B. subtilis*), cyan (Enterobacteriales), purple (5-sp), black (axenic). Data from vials containing flies reared with *P. putida*, which had characteristics of a fly pathogen, were omitted from these analyses.

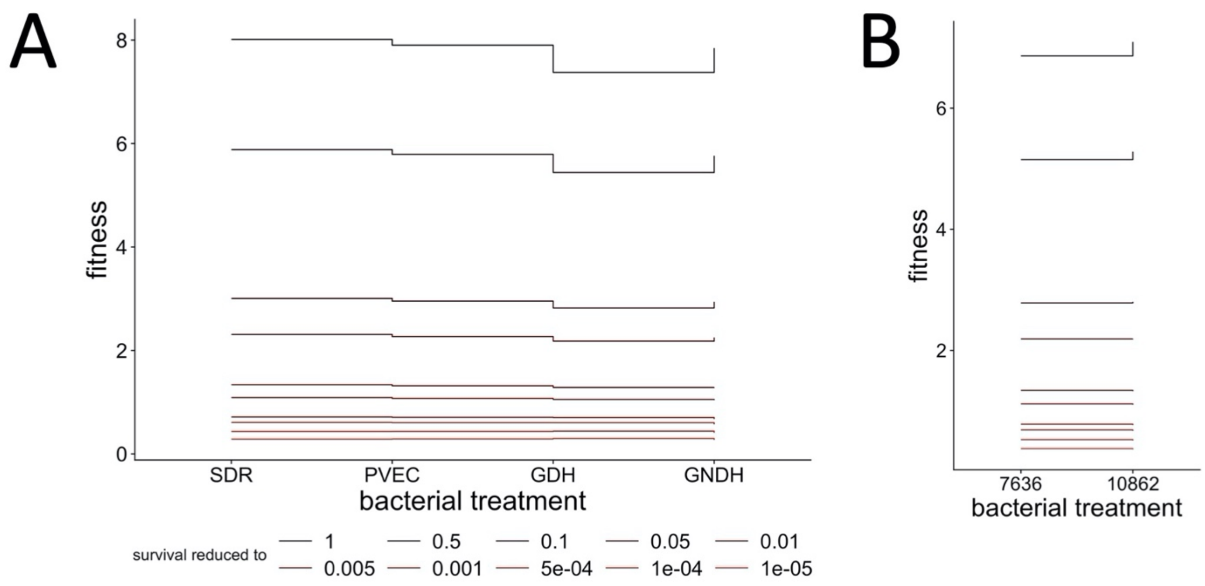

Figure S5. The effect on fly fitness of permuting fly survival rates under different bacterial treatments. To permute fly fitness, the same denominator was applied to the observed survival of the first age class across all of the different bacterial treatments. Each denominator value is shown as a distinct shaded line (see legend). The values are not continuous between treatments, but a single line facilitates visualizing the changes in rank order of fitness values conferred by the different bacterial treatments when permuted. Treatment names are from Table 2. No significant differences between bacterial treatments at each survival level were detected using a Kruskal-Wallis test.
